# Isolating Fungal Pathogens from a Dynamic Disease Outbreak in a Native Plant Population to Establish Plant-Pathogen Bioassays for the Ecological Model Plant *Nicotiana attenuata*

**DOI:** 10.1371/journal.pone.0102915

**Published:** 2014-07-18

**Authors:** Stefan Schuck, Arne Weinhold, Van Thi Luu, Ian T. Baldwin

**Affiliations:** Max Planck Institute for Chemical Ecology, Department of Molecular Ecology, Jena, Germany; The Ohio State University/OARDC, United States of America

## Abstract

The wild tobacco species *Nicotiana attenuata* has been intensively used as a model plant to study its interaction with insect herbivores and pollinators in nature, however very little is known about its native pathogen community. We describe a fungal disease outbreak in a native *N. attenuata* population comprising 873 plants growing in an area of about 1500 m^2^. The population was divided into 14 subpopulations and disease symptom development in the subpopulations was monitored for 16 days, revealing a waxing and waning of visible disease symptoms with some diseased plants recovering fully. Native fungal *N. attenuata* pathogens were isolated from diseased plants, characterized genetically, chemotaxonomically and morphologically, revealing several isolates of the ascomycete genera *Fusarium* and *Alternaria,* that differed in the type and strength of the disease symptoms they caused in bioassays on either detached leaves or intact soil-grown plants. These isolates and the bioassays will empower the study of *N. attenuata*-pathogen interactions in a realistic ecological context.

## Introduction

Pathogens from natural plant communities have largely been ignored for a long time, as Dinoor and Esched pointed out in their review in 1984, since phytopathologists have focused mainly on crop plants and plant ecologists have often failed to recognize the impact of phytopathogens on natural plant communities due to the general low incidence of disease in natural populations [1]. However, this changed as plant breeders soon started examining wild relatives of cultivated crop plants as potential sources of pathogen resistance traits [2]–[4] and wild plant pathosystems became valuable models for the study of plant-pathogen coevolution and evolutionary biologists and ecologists began to examine the influence of various biotic and abiotic factors, such as host and pathogen genetic diversity, habitat and community structures, and time scales on these important interactions [5]–[14].

Due to its synchronized germination from long-lived seed banks after sporadically occurring wildfires, *Nicotiana attenuata* typically forms monoculture-like populations exhibiting great genetic diversity and phenotypic plasticity [15]–[20]. These qualities make *N. attenuata* a very interesting model plant for studies of wild plant-pathogen interactions [21], [22]. However, in contrast to the tremendous repertoire of knowledge on *N. attenuata* ´s interactions with its native herbivores gained from the past two decades of field and laboratory research [23], reports on native phytopathogens of this native tobacco are scarce. One example is given by Rayapuram and Baldwin (2007) who observed disease symptoms caused by phytopathogenic *Pseudomonas* spp. on *N. attenuata* in nature [24]. Because native pathogens for bioassays on *N. attenuata* were lacking, common lab pathogen strains have been used, such as *Pseudomonas syringae* pv. *tomato* DC 3000 [25], the oomycete *Phytophthora parasitica* var. *nicotianae* and the ascomycete fungus *Fusarium oxysporum* f. sp. *tuberosi* [26]. The inferences from these studies with non-native pathogens may not be applicable to the real interactions that occur in nature [27]. Therefore the search for native *N. attenuata* pathogens has become more important in order to improve ecological relevance of the pathogen bioassay used to acquire ecologically relevant insights into the interaction of *N. attenuata* with its pathogens.

In the summer of 2011, populations of *N. attenuata* growing in the Great Basin Desert, Utah, USA suffered a dynamic disease outbreak likely caused by fungal pathogens. This study describes the dynamics of this fungal disease outbreak in a natural *N. attenuata* population, and how this outbreak was used to obtain native fungal isolates that were phylogenetically and biochemically characterized, and how candidate isolates with high pathogenicity potential were selected to establish laboratory bioassays with *N. attenuata*.

## Results

### The dynamics of disease symptoms a native *Nicotiana attenuata* population

**Figure 1:**
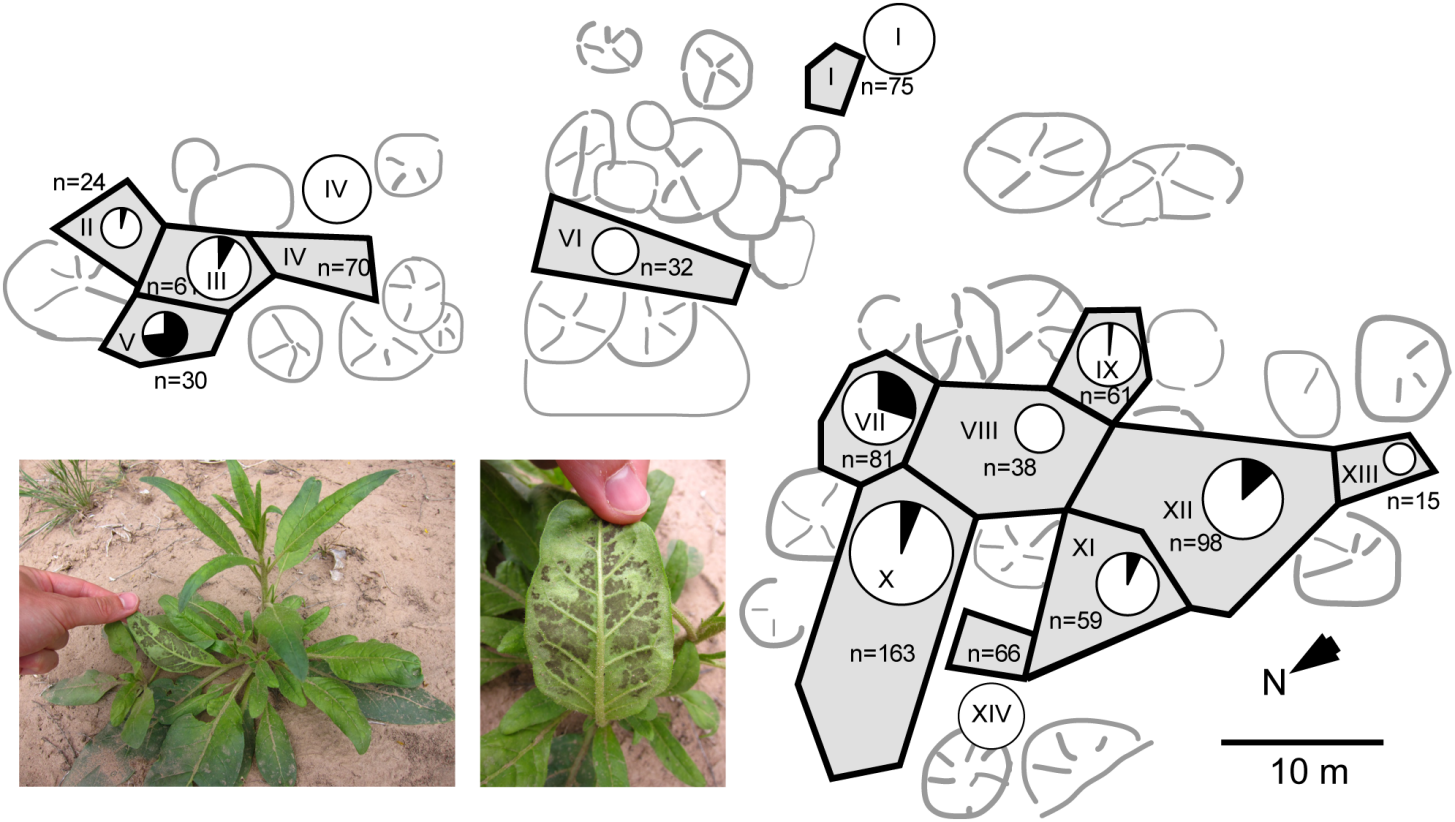
Schematic map illustrating the spatial distribution of diseased *N. attenuata* plants in a natural population. The plant number (n) is indicated for each section of the population (labeled I to XIV). The area of the circles represents the number of plants in each section. The black fractions represent the percentage of diseased plants in each section surveyed on the second monitoring (June 9^th^ 2011).

**Figure 2:**
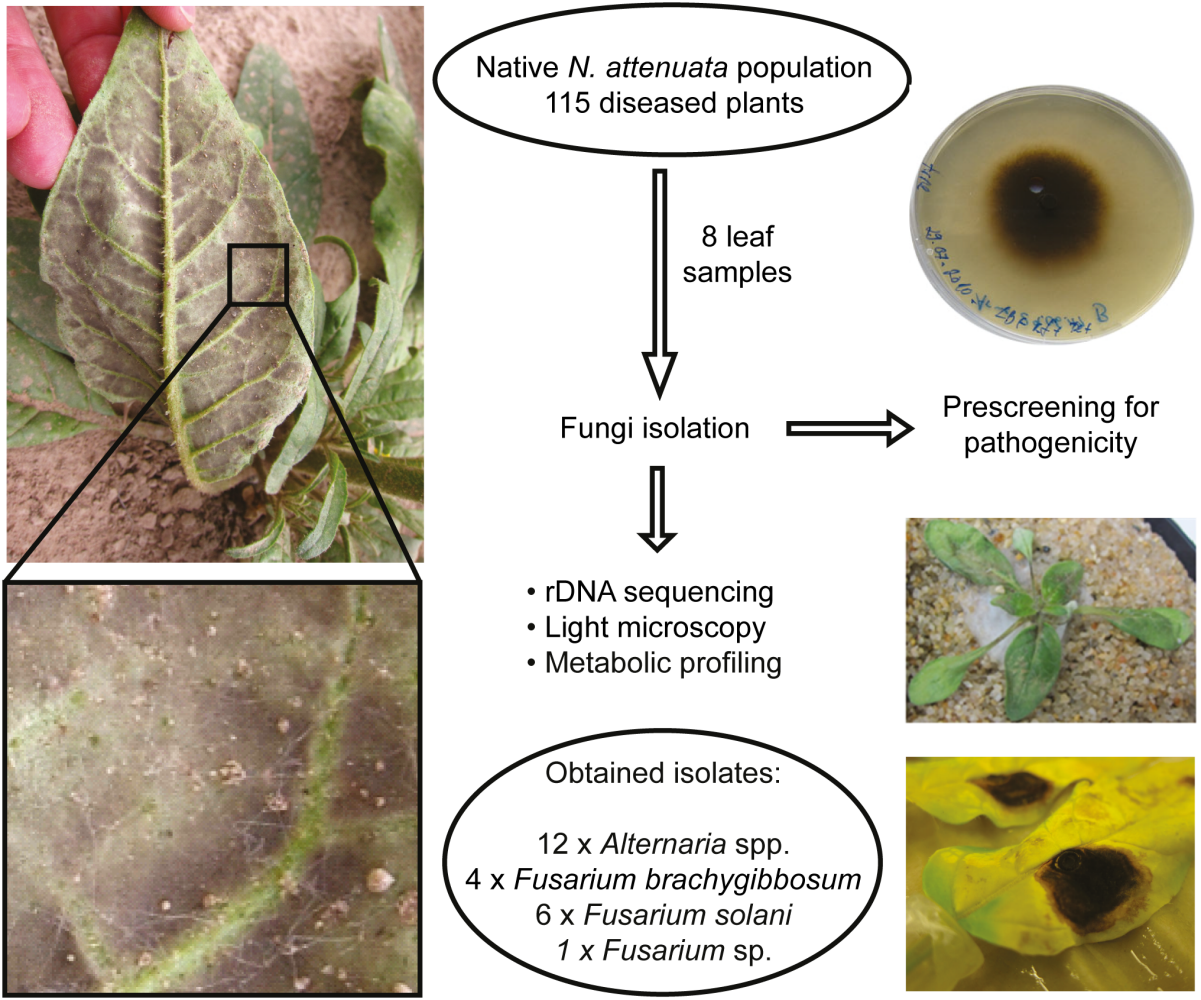
Work flow for the isolation of the native fungal pathogen isolates used in this study. In a native *N. attenuata* population plants suffered from disease symptoms (chlorosis, curly leaves and dark mycelium-like structures on the abaxial side, illustrated on the left). Leaf samples were taken from diseased plants for fungi isolation. Pathogenicity of the isolates was evaluated using different infection methods. Fungi, which were able to cause severe disease symptoms on plants were re-isolated from infected plant material and further characterized by rDNA sequencing, microscopy and their polar metabolite profiles, resulting in 12 *Alternaria* spp., 4 *Fusarium brachygibbosum*, 6 *F. solani* and one *Fusarium* sp. isolates.

During 2011 summer, a native *N. attenuata* population comprising 873 plants growing in an area of approximately 1500 m^2^ suffered a disease outbreak. The occurrence and strength of disease symptoms (dark spots on abaxial leaf surfaces which resembled fungal mycelium or sporangium-like structures shown in Figure 1 and 2, chlorosis, necrosis and curly leaves) were monitored during a 16 day-time interval (Table S1). The population was divided into 14 subpopulations (“sections”) according to habitat characteristics (grassland, shrubs, trees and areas free of *N. attenuata* plants) to better assess the disease dynamics. To be able to track the disease development for each individual plant, its position was marked in a schematic map of the respective section and assigned a number (Figure 1). All plants were categorized into four different disease stages as described in detail in the *Materials and Methods* section.

Even heavily diseased plants (disease stage category 3) with necrotic rosette leaves survived and attained the flowering stage. However, some of the diseased plants showed flower deformation with a shortened and bent corolla and protruding pistils. *N. attenuata* plants of different developmental stages (varying from young rosette stage to already flowering plants) occurred together in close association, but no stage-dependent disease prevalence was observed. Indeed, completely asymptomatic and heavily diseased plants of all plant developmental stages were found growing immediately next to each other. In some cases, the distance between diseased and asymptomatic plants was so small that their rosette leaves were touching each other.

Similar to the situation with neighboring plants, no disease symptom accumulation pattern could be observed on the scale of the different population sections. Sections with a relatively high percentage of diseased plants [section II: 41.7% (May 24^th^); section V: 66.7% (June 9^th^); section VII: 29.6% (June 9^th^)] or intermediate percentage [section III: 8.2% (June 9^th^); section X: 9.2% (May 24^th^); section XII: 13.3% (June 9^th^)] were not adjacent to each other and separated by nearly unaffected sections [section VIII: 2.6% (May 24^th^); section IX: 3.3% (May 24^th^); section XIII: 6.7% (May 24^th^)] or completely unaffected ones (sections I, IV, VI and XIV) (Figure 1 and Table S1). Only sections I and VI were spatially isolated from the other sections and entirely lacked diseased plants (Table S1).

With regard to the temporal pattern of disease symptom occurrence, 53.2% of the diseased plants recovered completely between the two surveys of the native *N. attenuata* population, while 6.5% of previously asymptomatic plants suddenly showed disease symptoms (Table S1). Remarkably, in section II, 90% of previously diseased plants recovered completely and no plant with progressing or newly occurring disease symptoms was found during the second survey (Table S1 and Figure S1). In contrast, in section VII, all plants were asymptomatic during the first survey and 29.6% suddenly showed disease symptoms during the second survey (Table S1 and Figure S1). In other sections (*e.g*. section X), the number of newly diseased (8.0%) and fully recovered (4.9%) plants was very similar and the overall number of diseased plants remained nearly unchanged (9.2% *vs.* 6.1%) (Table S1 and Figure S1). With regard to the entire population, the number of diseased *N. attenuata* plants increased slightly from 7.1% to 9.4%. Approximately 37.9% of all diseased plants showed a progression of disease symptoms, while 56.5% partly or fully recovered (Table S1). Overall, the disease outbreak turned out to be much more dynamic than could be inferred from only looking at the changes in the average values per section. In summary, diseased *N. attenuata* plants showed an astonishing capacity to fully recover.

### Classification of the native fungal isolates by ribosomal DNA sequence analysis

In order to identify and characterize potential causal agents responsible for the disease outbreak in native *N. attenuata* populations, eight leaf samples showing characteristic disease symptoms were collected and used to obtain fungal isolates. The isolation and characterization strategy is summarized in Figure 2. The fungal isolates were morphologically different and tested for their capacity to cause disease symptoms using the following inoculation procedure: the hypocotyls of 24 day-old *N. attenuata* plants were pricked with a needle before the application of actively growing fungus cultures to the wound sites as described in Bonaventure *et al*. [23]. This infection method provided reliable infections even for pathogens such as *Phytophthora parasitica* var. *nicotianae* and *Fusarium oxysporum* f. sp. *tuberosi* which are barely able to infect *N. attenuata* via spore suspension, and was therefore used to identify fungal pathogen candidates amongst the obtained native fungal isolates.

**Figure 3:**
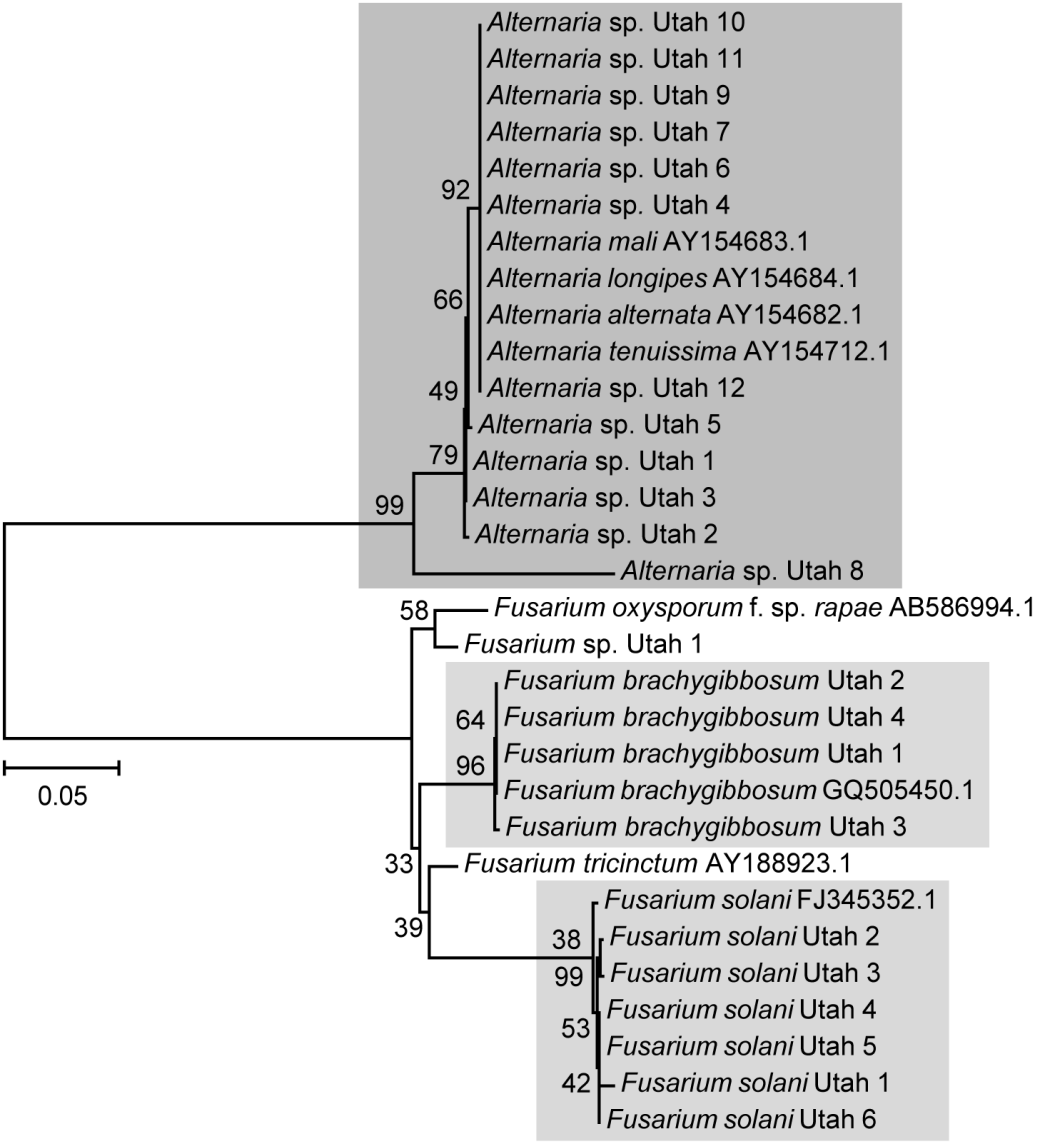
Phylogenetic relationship of native fungal pathogen isolates based on their rDNA sequence. The tree was constructed using the neighbor-joining method based on the sequences of ITS regions plus LSU using MEGA5 software. Sequences were aligned using ClustalW algorithm implemented in the MegAlign software. The numbers on the nodes indicate bootstrap scores in NJ analyses (1000 steps). The number on the scale indicates distance level with relative units.

23 native fungal isolates that caused disease symptoms were used for phylogenetic analysis (genomic DNA extraction and sequencing of their nuclear ITS-LSU rDNA region). The length of the amplified DNA region varied between the different fungal isolates from 1090 to 1156 bp, except for a sequence of a particular *Alternaria* isolate (*Alternaria* sp. Utah 6) being only 585 bp big due to poor quality PCR amplification. The rDNA sequences were aligned with ClustalW algorithm together with sequences of known reference strains, and a phylogenetic tree was constructed using the MEGA5 software (neighbor-joining tree using Kimura-2-parameter with 1000 bootstrap replications). The assignment to certain fungal species/genera via sequence similarity was performed according to criteria used by Bosshard *et al*. (2003) [28]. If the percentage of similarity of the query sequence and the reference sequence was 99% or above, the unknown isolate was assigned to the reference species. When percentage similarity was between 95 and 99%, the unknown isolate was assigned to the corresponding genus. Four native fungal isolates revealed 99% sequence homology to *Fusarium brachygibbosum* strain NRRL 34033 (GenBank accession number GQ505450.1) and were therefore assigned to *F. brachygibbosum* Utah 1 to 4 (Figure 3). Six native fungal isolates had 99% sequence similarity to *Fusarium solani* strain ATCC 56480 (GenBank accession number FJ345352.1) and were thus renamed to *F. solani* Utah 1 to 6 (Figure 3). The sequence of one native fungal isolate showed 98% sequence similarity to *Fusarium oxysporum* f. sp. *rapae* (GenBank accession number AB586994.1) and was therefore classified only at genus level as *Fusarium* spp. Utah 1 (Figure 3). Twelve other native fungal isolates revealed 99% sequence similarity to *Alternaria alternata* (GenBank accession number AY154682.1), *Alternaria tenuissima* strain IA287 (GenBank accession number AY154712.1), *Alternaria longipes* (GeneBank accession number AY154684.1) and *Alternaria mali* (GeneBank accession number AY154683.1), indicating that these twelve native fungal isolates belong to the genus *Alternaria*. However, they were indistinguishable at species level by rDNA sequence comparison (Figure 3).

### Chemotaxonomic characterization of the native fungal isolates

To support the fungal phylogeny data obtained by rDNA sequence analysis, we followed a polar metabolite profiling approach with the different native fungal isolates using liquid chromatography-time-of-flight-mass spectrometry (UPLC-ToF-MS). Fungal mycelium of the native pathogen isolates was scratched from the surface of 14 day-old fungal cultures and used for polar metabolite extraction. Negatively charged ions eluting from the column within a retention time of 3 to 481 s were analyzed by mass-spectrometry. Those having mass-to-charge values ranging from 107 to 1200 were selected for subsequent data analysis. Using the conditions mentioned above and after data processing, a total of 559 ions were identified. The cluster analysis of mass profiles with the MetaboAnalyst software was summarized as a dendrogram (Figure S2A) and showed a separation between the two fungal genera *Fusarium* and *Alternaria*. Within the genus *Fusarium*, a dichotomous clustering of *F. solani* and *F. brachygibbosum* isolates could be shown. The genus *Alternaria* clustered into three major subgroups, one containing *Alternaria* spp. Utah 4, 8 and 10, a second one with *Alternaria* spp. Utah 1, 11 and 12, and a third one with *Alternaria* spp. Utah 2, 3, 5, 6 and 9. To facilitate the graphical interpretation of the differences among the native fungal isolates, the data set corresponding to the differentially accumulating ions was subjected to a principal component analysis. The result is shown in Figure S2B for the separation of *Alternaria* spp. from *F. solani* and *F. brachygibbosum* isolates and in Figure S2C for the separation of the three different *Alternaria* isolate subgroups. Interestingly, the polar metabolite profiles of *Alternaria* spp. isolates revealed more within-taxon differences compared to both *Fusarium* species (*F. solani* and *F. brachygibbosum*) taken together (Figure S2B).

Since it is known that small-spored *Alternaria* species are hard to distinguish by rDNA sequence analysis [29], we also analyzed species-specific differences in the production of certain mycotoxins to further classify the native *Alternaria* isolates [30]–[34]. Common *Alternaria* mycotoxins are Alternariol (AOH), Alternariol monomethyl ether (AME), Altertoxin I (Alx I), Tentoxin (Ten) and Altenuene. AOH and Ten were found in *A. alternata* and *A. tenuissima*, but not in *A. mali* and *A. longipes* [31]–[33]. AME was found to be produced by *A. alternata*, *A. tenuissima* and *A. longipes*, but not *A. mali* [31]–[33]. Alx I is present in all four *Alternaria* species [31]–[33]. To identify these *Alternaria*-mycotoxins, mass-over-charge values and retention times of those compounds available at a public database were used to verify the UPLC-ToF-MS data for the presence of such compounds in the native *Alternaria* isolates [34]. AOH and Alx I were found to be produced by all native *Alternaria* isolates, as well as AME (except *Alternaria* sp. Utah 11) (Table S2). The presence of these metabolites indicates that all native *Alternaria* isolates belong either to *A. alternata* or *A. tenuissima.* However, Tentoxin typically produced by *A. alternata* and *A. tenuissima* could be detected for *Alternaria* spp. Utah 4 and 8 at only very low levels (Table S2). Even though this analysis was still not sufficient to exactly classify the native *Alternaria* isolates at the species level, it narrowed the choice to *A. alternata* and *A. tenuissima*.

### Morphological characterization of the native fungal isolates

**Figure 4:**
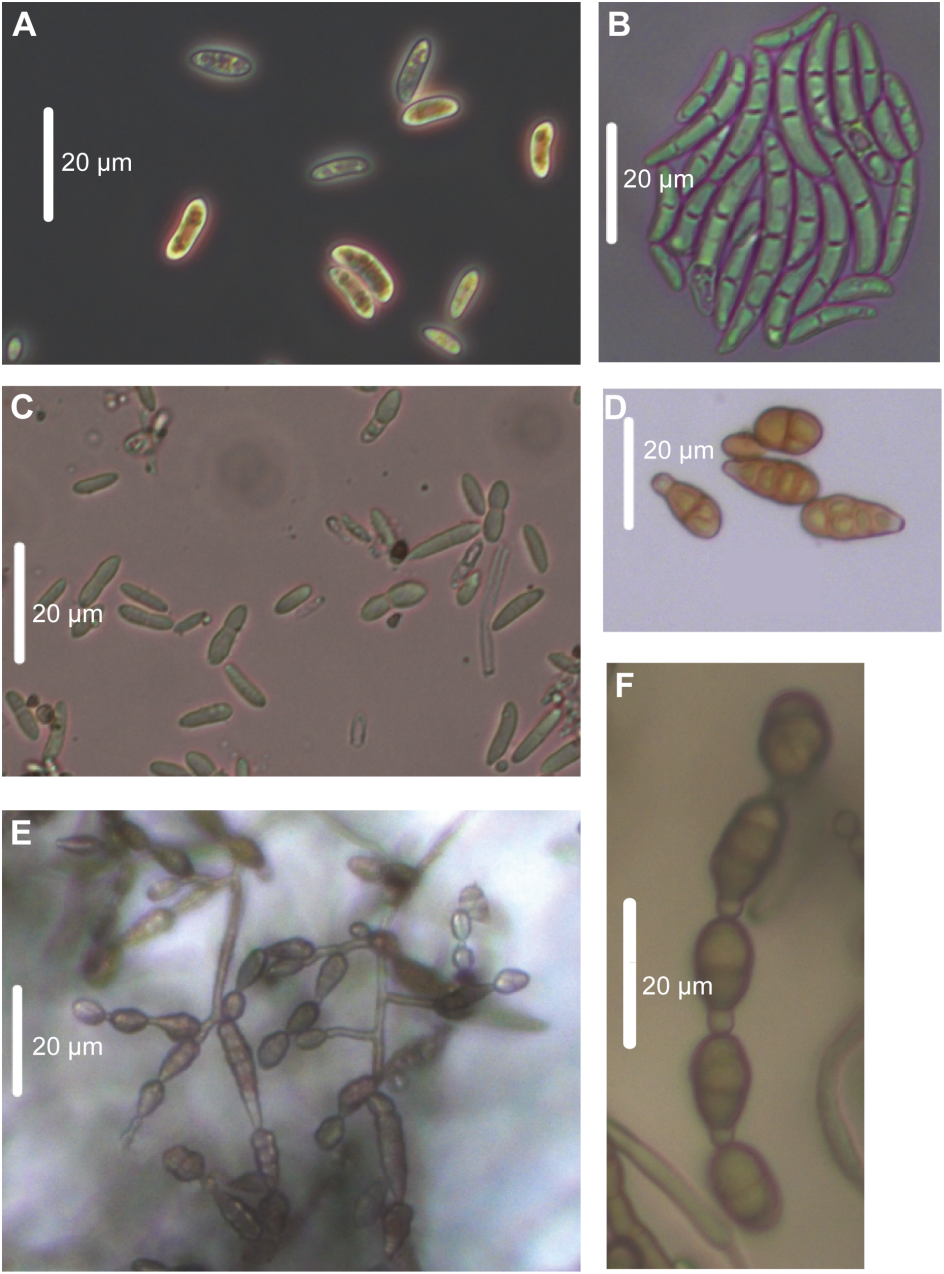
Morphology of native fungal pathogens observed by light microscopy. Conidia of 14 day-old fungal cultures grown for 14 days on PDA in the dark at room temperature. **A.** Microconidia of *F. solani* Utah 2. **B.** Macroconidia of *F. solani* Utah 4. **C.** Fungal spores of *F. brachygibbosum* Utah 4. **D.** *Alternaria*-typical conidia. **E.** *Alternaria* sp. Utah 10 sporulation clump and conidia. **F.** *Alternaria*-typical conidia chain. The white bars represent 20 µm.

In addition, the native fungal isolates were also characterized morphologically by analyzing the sporulation structures and conidia shapes of 14 day-old fungal cultures under the light microscope (Figure 4). Typical conidia shape, color, length and septum number were compared with reference conidia pictures of *Fusarium solani* published online at http://tolweb.org/Sordariomycetes/29050/2008.01.14. Isolates of *F. solani* Utah 1 to 4 could be confirmed with typical banana-shaped 3- to 5-septate macroconidia (Figure 4B). Oval-shaped 1- to 2-celled Microconidia were abundant (Figure 4A). In contrast to *Fusarium solani*, very little information is published about *F. brachygibbosum*, especially regarding its morphological characteristics. Spore structures of *F. brachygibbosum* Utah 1 to 4 were observed under the microscope as colorless spores, 1- to 2-celled with ellipsoid shape and 4 to 13 µm in length and 2 to 4 µm in width (Figure 4C). All native *Alternaria* spp. isolates had the conidia shapes characteristic of this genus, *i.e*. mostly ovoid with short conical beak at the tip (or beakless) (Figure 4D–F) [35], [36]. They were multicellular with several vertical and transverse septa (Figure 4D). Conidia color was pale brown. Interestingly, *Alternaria* sp. Utah 10 was showing the typical sporulation pattern of *A. alternata* with a single suberect conidiophore and an apical cluster of conidia chains (Figure 4E). Such conidia structures are typical for *A. alternata* with branching chains of small conidia separated by short secondary conidiophores (Figure 4F). This similarity between *Alternaria* sp. Utah 10 and *A. alternata* references published by de Hoog *et al.* (2000) [35] supports the assumption that *Alternaria* sp. Utah 10 might be a native *A. alternata* isolate.

### Development of bioassays using *N. attenuata* and its native fungal pathogens

**Figure 5:**
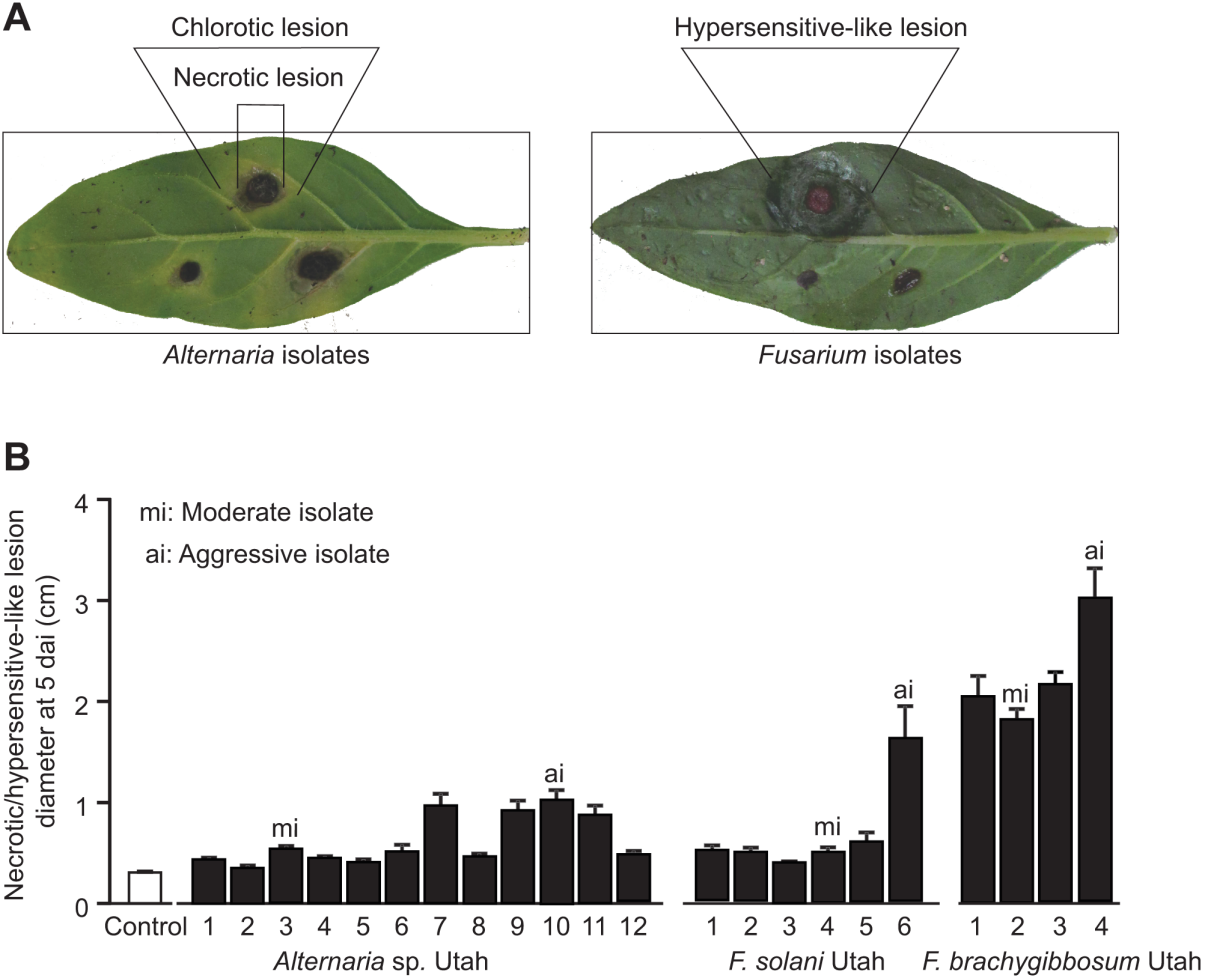
Evaluation of fungal pathogenicity using detached leaf assay. **A.** Disease symptoms caused by *Alternaria* and *Fusarium* on *N. attenuata* detached leaves. Agar plugs containing fungus culture were placed on detached leaves of *N. attenuata* and incubated at high humidity. Detached leaves incubated with pure PDA plugs were used as control. 12 biological replicates were used for each fungal isolate. Photos were taken at 3 and 5 days after inoculation for *Fusarium* and *Alternaria* isolates, respectively. **B.** Necrotic lesion diameters of *Fusarium-* and *Alternaria*-inoculated leaves at 5 days after inoculation (dai). The most aggressive isolates (ai) caused the largest necrotic/hypersensitive-like lesion on detached *N. attenuata* leaves while the moderate isolates (mi) caused visible necrotic/hypersensitive-like lesions only slightly larger than the diameter of pure agar plugs (control).

To assess the virulence of the individual fungal isolates on *N. attenuata* plants, a detached leaf assay was performed by placing agar plugs with fungal cultures on excised *N. attenuata* leaves (Figure 5). The differences in virulence of the native fungal isolates were evaluated based on average diameters of chlorotic, necrotic and hypersensitive-like lesions. Native *F. brachygibbosum* isolates were the first ones to cause visible symptoms in form of hypersensitive-like lesions after three days of incubation (Figure 5A). The lesions expanded rapidly from the inoculation points and started to overlap each other already two days later. Chlorotic lesions were not detected for *F. brachygibbosum* isolates. Only *Alternaria* spp. Utah 10 and 11 could induce chlorotic lesions already three days after inoculation, but after five days of incubation all detached *N. attenuata* leaves inoculated with native *Alternaria* isolates started to show chlorosis and necrosis (Figure 5A). Leaves inoculated with native *F. solani* developed chlorosis and necrosis at four to five days after inoculation. For leaves inoculated by native *Alternaria* and *F. solani*, necrotic lesions were measured at five days after inoculation and were used to evaluate the aggressiveness of the fungal isolates (Figure 5B). Among the twelve *Alternaria* isolates, *Alternaria* sp. Utah 10 which caused the largest necrotic lesions (1.03±0.09 cm diameter) appeared to be the most aggressive isolate, followed by *Alternaria* spp. Utah isolates 7, 9 and 11. *Alternaria* sp. Utah 3 was assigned as a moderate isolate since it was able to cause visible necrotic lesions which were significantly (*P* = 0.039) larger (0.54±0.02 cm) than the pure agar plug diameter from control leaves (0.31±0.01 cm), but significantly less aggressive than *Alternaria* spp. Utah isolates 7, 9, 10 and 11 (*P*<0.001) (Figure 5B). The necrotic lesions caused by *Alternaria* spp. Utah 1, 2, 4, 5, 6, 8 and 12 were not significantly different from the diameter of the pure agar plug (Figure 5B). Therefore these fungal strains were defined as being less aggressive isolates. Among the six native *F. solani* isolates, *F. solani* Utah 6 turned out to be an aggressive isolate since it caused the largest necrotic lesion (1.61±0.31 cm) at five days after inoculation compared to controls and the other native *F. solani* isolates (*P*<0.001) (Figure 5B). *F. solani* Utah 1, 2, 3, 4 and 5 were not significantly different from controls and therefore considered as less aggressive isolates (Figure 5B). Interestingly, *F. brachygibbosum* Utah caused hypersensitive-like lesions which were significantly bigger compared to the necrotic lesions caused by native *Alternaria* (*P*<0.001) and *F. solani* isolates (*P* = 0.002) (Figure 5B). At five days after inoculation on detached *N. attenuata* leaves, *F. brachygibbosum* Utah 4 was the most aggressive isolate with hypersensitive-like lesion diameters of 3.04±0.28 cm, followed by the rather moderate isolates *F. brachygibbosum* Utah 1 to 3 (Figure 5B). This experiment helped to assess the virulence of each native fungal isolate necessary for choosing the appropriate candidates for further bioassays.

**Figure 6:**
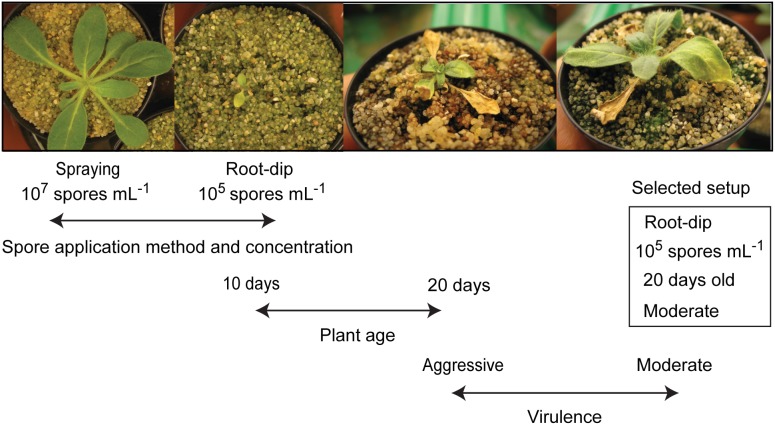
Testing different infection setups using *F. solani* spore suspension and intact *N. attenuata* plants. Two different infection methods (spore-spraying and root-dip), three different spore concentration (10^7^, 10^6^ and 10^5^ spores mL^−1^), two different plant ages (10 and 20 days-old) and two different virulence levels (aggressive and moderate *F. solani* Utah isolate) were tested with intact soil-grown plants.

To be able to study the interaction between *N. attenuata* and its native pathogenic fungi in a more realistic way (*i.e*. mimicking natural infection conditions as much as possible), an infection assay using intact *N. attenuata* plants and fungal spore suspension needed to be developed for all three native fungal pathogen species. Based on the pathogenicity evaluation result from the detached-leaf assay, the most aggressive and a “moderate” isolate of native *F. brachygibbosum*, *F. solani* and *Alternaria* spp. were tested in intact-plant infection assays in order to identify the isolate with the most appropriate virulence for each native pathogen species. *F. brachygibbosum* Utah 2 was used as a moderate *F. brachygibbosum* isolate because it had the smallest necrotic lesion diameter (1.80±0.09 cm) compared to its conspecifics. For *F. solani* no moderate isolate was available, therefore the less virulent *F. solani* Utah 4 strain was randomly selected. Root-dip and spore-spraying methods were chosen to reflect the natural infection process of soil-borne and aerial pathogens as it would occur in the native habitat of *N. attenuata*. Development of disease symptoms on infected plants was observed and recorded for 20 days after inoculation by estimating the percentage of chlorosis and necrosis compared to the total leaf area. In contrast to diseased plants in natural *N. attenuata* populations, dark-spotted abaxial leaf sides, leaf curling and changes in flower morphology could not be observed within the 20 days after inoculation. Different plant ages (10 to 20 days after germination) were tested with regard to their suitability for infection. The work flow for the development of a suitable infection assay with native *F. solani* is described in detail in Figure 6 as an example of the workflow used. Inoculation of young *N. attenuata* seedlings (10 day-old) by spraying spore suspension of *F. solani* Utah 6 did not result in any diseased plant even at the highest spore concentration tested (10^7^ spores mL^−1^) (Figure 6). This spore application method proved to be an ineffective inoculation procedure also for all of the other fungal isolates tested. In contrast, dipping seedling roots into spore suspension resulted in heavily diseased plants even with the lowest tested *F. solani* spore concentration (10^5^ spores mL^−1^), indicating that the root-dip method was very effective for inoculation. However, inoculation of very young seedlings (10 days-old) resulted in disease symptoms that were too severe for experimentation and did not reflect the disease severity observed in the natural population. Therefore older plants (20 days-old) were also tested, but disease symptoms were still too severe in case of the aggressive *F. solani* Utah 6. *F. solani* Utah 4 caused much less severe and yet clearly visible disease symptoms, and provided the best bioassays reflecting a near-natural infection process. The infection assays for the two other native fungal species were tested and optimized in a similar manner. Inoculation with the most aggressive native *F. brachygibbosum* isolate (*F. brachygibbosum* Utah 4) to 20 day-old plants using the root-dip method did not result in any visible disease symptoms. Diseased plants could be obtained only when very young seedlings (10 day-old) were inoculated with the highest spore concentration (10^7^ spores mL^−1^). *F. brachygibbosum* was barely infective when used on intact *N. attenuata* plants, which is in good agreement with the strong hypersensitive-like lesions on detached leaves pointing to a high degree of plant resistance to this pathogen [37]. A root-dip (10^5^ spores mL^−1^) bioassay using 12 day-old *N. attenuata* seedlings and the most aggressive native *Alternaria* isolate (*Alternaria* sp. Utah 10) proved to be most appropriate experimental setup for this native fungal pathogen species.

## Discussion

### Disease dynamics in a native *N. attenuata* population

Within only 16 days, disease symptom development in a native *N. attenuata* population appeared very dynamic, since more than half of all diseased plants recovered completely, while at the same time many previously asymptomatic plants started to show symptoms (Figures 1; Figure S1; Table S1). No clear spatial pattern was observed in the occurrence of disease symptoms within the native *N. attenuata* population at the two survey time points. The sections with high recovery rate (sections II, V and X) and sections with a high abundance of newly diseased plants (sections V, VII, X and XII) were spatially disjunct and distributed over almost the complete population, being separated by more or less unaffected population sections (Figure S1 and Table S1). The lack of a spatial pattern was also observed within population sections, since diseased plants were usually not clustered and surrounded by completely asymptomatic plants. Micro-spatial heterogeneity of spore density within the soil could be a potential explanation for the distribution of diseased plants [38]. However, our data seems to support another potential explanation for this phenomenon: native *N. attenuata* populations typically harbor a great deal of genetic diversity which could lead to high phenotypic plasticity even on very small spatial scale, likely due to their long-lived seed banks [16]–[18], [20], [39]. In contrast to domesticated crop plants, wild plant populations are genetically diverse and based on each plant´s individual genotype, the ability to cope with fungal pathogen attack may differ within a native *N. attenuata* population [16]–[18], [21], [22], [39]. This genetic variability within a population could augment the phenotypic plasticity that occurs within individuals and could explain the coexistence of individuals with different defense strategies, as has been shown for plant-herbivore interactions [16]–[18], [39].

Plant defense against pathogens implies signaling processes mediated by phytohormones such as salicylic and jasmonic acid, and depending on the individual plant´s genotype and this phytohormone signaling response to pathogen attack may vary even among neighboring plants [16], [18], [40]. Disease symptoms could be observed within a wide range of different plant developmental stages in the native *N. attenuata* population, suggesting a stage-independent disease prevalence. However, age-related resistance as reported for *Nicotiana benthiamana* to the oomycete pathogen, *Phytophthora infestans,* is not uncommon [41]. Indeed, the results from our bioassays suggest an age-related resistance of *N. attenuata* to native *Alternaria* spp., *Fusarium solani* and *Fusarium brachygibbosum* as well, since high infection efficiency was only observed when young plants (10 to 20 days after germination) were exposed to the pathogen. Therefore we speculate that plants from the native *N. attenuata* population were probably infected already at a very early developmental stage before the monitoring of disease symptoms started, and developed symptoms after a latency period which could vary among individuals. In addition to the proposed variability in this latency period, differences in the time of germination together with long-lasting conditions favorable for pathogen infection could also provide a plausible explanation for the wide range of plant developmental stages that were affected.

Such disease-promoting conditions could have been created by the weather situation a few weeks prior the first survey of diseased plants: for several days in a row, strong winds were blowing and sporadic rainfalls (sometimes in association with thunderstorms) created an unusually cool and moist environment in the native *N. attenuata* habitat in 2011. Even in the relatively dry environment of the Great Basin Desert, fungal fruiting bodies emerged out of the sandy soil surface between the native *N. attenuata* plants. The wind blew around a lot of sand and dust particles which could have caused small wounds on the plant surface and created potential entry sites for all sorts of pathogens. Such environmental conditions are thought to be conducive for infection for a variety of fungal pathogens [42]–[46].

Despite a population-wide slight increase in disease incidence and symptom severity (Table S1), none of the plants were observed to die from the disease. Together with the astonishing high capacity to recover from all symptoms, this suggests that the plants harbor effective and adaptive defense responses against the causal disease agent(s). This property makes *N. attenuata* an interesting plant species to study plant defense against pathogens. While the disease outbreak did not result in any mortality within the monitored native *N. attenuata* population, it clearly seriously reduced the Darwinian fitness of diseased plants by reducing growth (leaf damage) and reproduction (flower deformation).

### A complex of *Alternaria* and *Fusarium* species as the causal agent for the disease outbreak observed in nature

From the wild *N. attenuata* population monitored during the disease outbreak, leaves showing typical symptoms were used to obtain native fungal isolates as potential causal disease agents (Figure 2). These isolates were identified as *Alternaria* spp., *Fusarium solani* and *F. brachygibbosum* (Figure 3), known from the published literature to be phytopathogens that occur in arid environments [47]–[51]. Ribosomal DNA sequence analysis commonly used for fungal phylogenetics was sufficient to distinguish the *Fusarium*, but not the *Alternaria* isolates at the species level [29], [52]. To support fungal species/genus identity of the native isolates and to further narrow the number of possible species for the *Alternaria* isolates, actively growing fungus cultures were characterized by light microscopy (Figure 4) and their polar metabolite profiles (Figure S2 and Table S2). Both methods were consistent with the results of the rDNA sequence analysis and pointed to an assignment of *Alternaria* spp. Utah 4, 8 and 10 to *Alternaria alternata* due to the sporulation pattern (Figure 4E) and the similarity of their polar metabolite profiles (Figure S2C). *Alternaria* spp. Utah 1–3, 5, 6, 9, 11 and 12 could be theoretically assigned either to *A. alternata* or *A. tenuissima* due to the presence of Alternariol, a metabolite indicative of the two *Alternaria* species in all of these native *Alternaria* isolates (Table S2) [30].

The native fungal isolates were originally picked from diseased *N. attenuata* samples (seedlings or leaf cuttings) according to their culture morphology. As a consequence, all obtained isolates differed already morphologically from each other, even if they later turned out to be closely related according to their rDNA sequence data (Figure 3). These morphological differences on culture medium were also reflected in virulence differences as shown with a bioassay using detached *N. attenuata* leaves (Figure 5). Natural variability of a certain fungal species in virulence is known to occur on a very small spatial scale (*i.e*. within a single plant) [14]. Co-infection events with different pathogen strains belonging to the same species might positively influence virulence evolution, transmissibility and the maintenance of pathogenicity polymorphism in local pathogen populations [14]. This would also explain why so many different *Alternaria* spp., *F. solani* and *F. brachygibbosum* isolates could be found in only a few leaf samples from diseased *N. attenuata* plants. Application of only a single native fungal isolate could not reproduce the full spectrum of symptoms observed on diseased *N. attenuata* plants in nature. Plant infection by multiple fungal isolates/species (either simultaneously or in a certain sequential order) might be necessary to mimic all symptoms observed in the natural *N. attenuata* population in 2011. We hypothesize that a combination of *Alternaria* and *Fusarium* species could form a disease complex leading to *N. attenuata* superinfection events in nature. Indeed, it is known for several plant diseases, *e.g.* pea root rot and hazelnut brown apical necrosis, that both of these fungal genera typically co-occur in such fungal disease complexes [53]–[55].

### Establishment of bioassays for *N. attenuata* using its native fungal pathogens

As mentioned in the introduction, there was an increasing demand to find native pathogens for *N. attenuata* in order to improve pathogen bioassay quality and to study the interaction of *N. attenuata* with its pathogens in nature. This work yielded a large repertoire of native fungal pathogen isolates differing largely in their virulence, and enables *N. attenuata* researchers to choose the pathogen most appropriate for their experiments from this resource. In contrast to other common lab pathogens previously used for bioassays with *N. attenuata* [25], [26], the native fungal isolates are known to interact with *N. attenuata* in nature and therefore are particularly interesting for those experiments which aim to draw inferences to natural infections. To establish such a near-natural pathogen bioassay setup for *N. attenuata*, we used fungal isolates which can easily infect entire plants via spore suspension. Such bioassays were successfully developed for all three native pathogen groups (*Alternaria* spp., *F. solani* and *F. brachygibbosum*) by dipping the roots of young *N. attenuata* plants into fungal spore suspension (Figure 6). Small wounds at the roots generated by removing *N. attenuata* plants from their growth medium could have served as pathogen entry sites and facilitated plant infection by the native fungal pathogens. This root-dip method was sufficiently efficient to ensure infection and allowed the plants to gradually develop disease symptoms (chlorosis and necrosis) over a long time period. This gradual disease progression might be especially useful for pathogen bioassays with *N. attenuata* which require a long-term observation of the plant-pathogen interaction, *e.g*. in tripartite-interaction experiments involving a rather slow developing herbivore species. Further studies could also use the obtained fungal isolates for co-infection experiments to address the following questions: could the symptoms observed in natural *N. attenuata* populations in 2011 be explained by a disease complex comprising *Alternaria* and *Fusarium* species? How large is the genetic variability in native *N. attenuata* populations with regard to the susceptibility to these pathogens? How does *N. attenuata* respond to the attack by native *Alternaria* and *Fusarium* species, *e.g.* on the level of phytohormone signaling and defensive secondary metabolite production? Obviously this plant species possesses defense responses which are efficient enough to enable many diseased plants to fully recover from all symptoms in nature. Since it is known that *Alternaria* and *Fusarium* species even possess pathovar-characteristic supernumerary chromosomes enabling infection of a distinct host range, it appears likely that the obtained native fungal isolates are specialized on *N. attenuata* (and possibly related species) as well [56]–[58]. In addition, the obtained fungal isolates might well be locally adapted to the surveyed *N. attenuata* population due to the sympatric occurrence of host and pathogen species [8], [13]. For the native fungal isolates, the degree of host specialization and the extent of local adaption remain to be tested in future. This work will hopefully encourage other plant scientists still lacking a native pathosystem for their model plant to make similar attempts to search for ecologically relevant pathogens as well.

## Materials and Methods

### Ethics statement

*N. attenuata* Torrey ex S. Watson seeds were originally collected from a wild accession on private property (DI ranch) in south western Utah in 1988. Ian Baldwin had permission from the owner, Herbert Fletcher to do this collection, and Herbert Fletcher has since deceased. The monitored native population of *N. attenuata* plants was located on private property of the Lytle Ranch Preserve (N 37.1427 W 114.0229), 45 miles northwest of St. George, Utah, USA, which is run by the Brigham Young University of Utah. None of the work was done with endangered species

### *Nicotiana attenuata* cultivation

*N. attenuata* seeds of a 31^st^ inbred generation were cultivated as previously described [59]. Seeds were sterilized for 5 min in 5 mL aqueous solution containing 0.1 g dichloroisocyanuric acid (DCCA: Sigma, St. Louis, MO, USA), supplemented with 50 µL of 0.5% (v/v) Tween-20 (Merck, Darmstadt, Germany). Seeds were washed three times with sterile water before incubation for 1 h in 5 mL sterile liquid smoke (House of Herbs, Inc.; Passaic, New Jersey, USA) 50-fold diluted in deionized water and supplemented with 50 µL of 0.1 M gibberellic acid (Roth, Karlsruhe, Germany). After this treatment, seeds were washed three times with sterile water and 25 seeds were transferred individually to a petri dish containing germination medium [Gamborg’s B5 medium with minimal organics (Sigma) and 0.6% (w/v) phytagel (Sigma)]. Plates were maintained in a growth chamber (Snijders Scientific, Tilburg, the Netherlands) at 26°C for 16 h light (155 µmol s^−1 ^m^−2^) and 24°C for 8 h dark.

Ten day-old *N. attenuata* seedlings, which were used for entire-plant bioassays with fungal isolates, were transferred to TEKU plastic pots (Pöppelmann GmbH & Co. KG, Lohne, Germany) with sterile sand and grown in climate chambers with a day/night cycle of 16 h (20°C) and 8 h (20°C) under supplemental light from Master Son-T PIA Agro 400 and a relative humidity of 60%. After 10 days, early rosette plants were transferred into 10 cm-diameter pots and grown in the same climate chambers. Each plant was fertilized with 50 mL of Ca(NO_3_)_2_×4 H_2_O and 0.4 g L^−1^ Flory B1.

Plants used for detached leaf assays were transferred after 10 days in the growth chamber into the TEKU plastic pots with Klasmann plug soil (Klasmann-Deilmann GmbH, Geesten, Germany). After 10 days in TEKU pots, seedlings were transferred to 1 L pots in soil [0.75 g Superphosphate, Multimix 14∶16–18 (Haifa Chemicals Ltd., Haifa Bay, Israel), 0.35 g MgSO_4_×7 H_2_O (Merck KGaA, Darmstadt, Germany), 0.05 g Micromax (Scotts Deutschland GmbH, Nordhorn, Germany) per 1 L Fruhstorfer Nullerde (Hawita GmbH, Vechta, Germany)]. Fertilization was done also by flood irrigation with addition of 3.0 g Borax (Nic Sosef BV, Honselersdijk, Netherlands) on the day of potting, 2.0 g Borax and 20 g Peters Allrounder 20∶20∶20 (Scotts International, Heerlen, Netherlands) from day seven on, 1.0 g Borax, 40 g Peters Allrounder from day 14 on and 25–30 g Peters Allrounder from day 21 on, each to a 400 L watering tank. From day one up to day five, plants were watered with 300 mL and later with 100 mL per day and grown in the glasshouse at 26–28°C under 16 h supplemental light from Master Sun-T PIA Agro 400 or Master Sun-T PIA Plus 600 W Na lights (Philips, Turnhout, Belgium).

### Disease symptom monitoring of the native *N. attenuata* population

During the field season in 2011, plants in several native *N. attenuata* populations had been discovered showing symptoms apparently caused by a fungal disease. To assess the ecological impact of the disease outbreak on this plant species, a particular *N. attenuata* population on the Lytle Ranch Preserve comprising 873 plants distributed over an area of approximately 1500 m^2^ was monitored for a population-wide screening of disease incidence and symptom severity. The area was divided into 14 subpopulations ( = “sections”) of 15 to 163 plants according to habitat properties (*e.g.* grassland, shrubs, trees and larger areas without any *N. attenuata* plant). To mark the sections, colored flags were put into corners and connected via colored woolen threads. Then schematic maps (not absolutely to scale) were drawn by hand to record the spatial distribution. In each section the positions of the individual plants were assigned with a number to track disease development for each plant position within the population. Afterwards all plants were screened with regard to the occurrence and strength of disease symptoms. In more moderate cases diseased plants appeared almost asymptomatic at first glance, however, when inspected more carefully, showed dark spots at the abaxial leaf side having a rough surface (most likely fungal mycelium plus sporangia; Figures 1 and 2). Such plants were classified into disease stage category 1. In addition, some plants turned chlorotic and had curly leaves and were therefore classified into disease stage category 2. In severe cases, plants developed necrotic lesions and were classified into disease stage category 3. The percentage of chlorotic and necrotic leaf area was estimated for each plant relatively to its overall leaf area. This population-wide screening for disease symptoms from May 24^th^ 2011 was repeated 16 days later on June 9^th^ 2011 in order to see how disease symptoms develop over time at the individual *N. attenuata* plants. Completely asymptomatic plants were categorized into “disease stage 0”.

### Fungi isolation and cultivation

In order to culture potentially pathogenic fungi from diseased *N. attenuata* plants, eight leaf samples clearly showing disease symptoms were brought from the above mentioned native *N. attenuata* population to the Max Planck Institute, Jena, Germany. The process of fungi isolation is illustrated in Figure 2. Sampling was performed from freshly diseased plants not randomly distributed across the population sections. Leaf samples came from sections III, VII and VIII (majority from sections VII and VIII) (Figure 1). Fungi were isolated using different strategies. Two leaf samples were thoroughly ground with a pestle and mortar and diluted in 5 mL sterile deionized water, and 20 µL of the leaf suspension was applied onto 10 day-old *N. attenuata* seedlings growing on Gamborg B5 medium. A few days after inoculation, most of the seedlings started to turn chlorotic and wilting. Diseased seedlings were either surface-sterilized [60 s in 0.1% (w/v) DCCA in deionized water with three subsequent washes in sterile deionized water] or just rinsed in sterile deionized water. In addition, six leaf samples were cut with a sterile scalpel into approximately 5 mm big pieces and the obtained leaf cuttings were either surface-sterilized (two minutes in 0.1% DCCA (w/v) in deionized water with three subsequent washes in sterile deionized water) or just rinsed in sterile deionized water. Surface-sterilized and non-sterilized leaf cuttings/seedlings were then transferred onto various media [Luria-Bertani agar containing 25 mg L^−1^ Rifampicin and 5 mg L^−1^ Tetracyclin, Kaefer medium [60] agar and potato dextrose agar (PDA; Fluka Analytical, Steinheim, Germany)] and incubated at 25°C in the dark until fungal hyphae emerged from the plant tissue. Based on morphological differences (color and shape of mycelium) fungal cultures were picked and cultured on PDA plates. The obtained morphological different fungal isolates were inoculated to 24 day-old *N. attenuata* plants as described in Bonaventure *et al*. (2011) for an initial pathogenicity test [26]. In brief, 14 day-old fungal cultures were transferred to the hypocotyl of sand-grown plants after pricking it with a sterile syringe needle to facilitate a potential infection. Those fungal isolates which were able to cause plant disease symptoms within seven days were chosen for rDNA sequencing, morphological and chemical characterization and further bioassays (Figure 2). The *Alternaria* and *Fusarium* fungal isolates used in this study were re-isolated from infected *N. attenuata* plants from time to time and confirmed by morphological comparison to the originally isolated and characterized fungal cultures (mycelium growth pattern, structure and color, and conidia-shape by light microscopy).

### Phylogenetic analysis of fungal isolates based on their rDNA-ITS-LSU sequence

Fungal DNA was extracted from mycelium for PCR amplification using the following protocol: 14 day-old fungal culture grown on PDA plates was used to extract total fungal DNA according to a slightly modified DNA extraction protocol using the Cetyl trimethylammonium bromide (CTAB) method as described by Ben Bubner (2004) [61]. Approximately 300 mg fungal mycelium material was scraped directly from the surface of the plates and immediately frozen in liquid nitrogen. The samples were ground with a 2000 Geno*/*Grinder machine (SPEX Certi Prep, Metuchen, New Jersey, USA) and kept frozen until extraction with liquid nitrogen. Extraction was performed with 800 µL preheated (65°C) buffer [2% (w/v) CTAB, 100 mM Tris pH 8.0, 20 mM EDTA pH 8.0, 1.4 mM NaCl, 1% (w/v) Polyvinylpyrrolidone M_r_ 40000, 1% (v/v) 2-mercaptoethanol] and 500 µL of chloroform/isoamyl alcohol (24∶1; v/v). After being mixed and centrifuged, the supernatant was supplemented with 0.1 volume 10% (w/v) CTAB and further cleaned up using again phase-separation with one volume of chloroform/isoamyl alcohol (24∶1; v/v). The nucleic acids in the resulting supernatant were precipitated with an equal volume of ice cold isopropanol. After rehydrating the pellet in 500 µL high salt TE (10 mM Tris pH 8.0, 1 mM EDTA pH 8.0, 1 M NaCl, 100 ng µL^−1^ RNase A; Roth, Karlsruhe, Germany), the solution was incubated at 37°C for 15 min. Following a phase separation step with 500 µL chloroform/isoamyl alcohol (24∶1; v/v), the supernatant was taken for DNA precipitation by adding 1 mL ice cold isopropanol. The pellet resulting from centrifugation was washed with 80% (v/v) ethanol and rehydrated in 50 µL Milli-Q water (Milli-Q Advantage A10, Millipore, Massachusetts, USA). OD_260_ measurements on a Biophotometer (Eppendorf, Hamburg, Germany) indicated concentrations between 0.05 to 0.25 µg µL^−1^.

An approximately 1200 bp long genomic DNA sequence comprising the internal transcribed spacer (ITS) 1 and 2 regions, the 5.8S rDNA and part of the large ribosomal subunit (LSU) gene was amplified by PCR from fungal genomic DNA samples using the primer pair ITS1-F (5′-TTGGTCATTTAGAGGAAGTAA-3′) and TW13 (5′-GGTCCGTGTTTCAAGACG-3′) [52], [62]. PCR reagents were obtained from Fermentas GmbH, Germany. The PCR mix contained 0.5 µL of a 10 mM deoxynucleoside triphosphate mix, 0.5 U of *Taq* DNA polymerase, 0.2 µM of each primer, 1 µL 1∶10-diluted genomic DNA extract, 25 mM MgCl_2_ and 2.5 µL *Taq* buffer (10x) in a total volume of 25 µL supplemented with the appropriate amount of Milli-Q water. PCR amplification was conducted using a Mastercycler gradient thermocycler (Eppendorf, Hamburg, Germany) with the following program: initial denaturation for 3 min at 95°C, followed by 20 cycles of denaturation for 30 s at 95°C, annealing for 30 s at 60°C and elongation for 3 min at 72°C, followed by a final extension step at 72°C for 10 min. Amplification products were visualized and quantified using horizontal gel electrophoresis and a 1% (w/v) agarose gel (Sigma-Aldrich Chemie GmbH, Hamburg, Germany) in 0.5x Tris-borate-EDTA-running buffer and ethidium bromide staining.

PCR products were sequenced from both strand ends using the same primers as described above. BigDye terminator cycle sequencing (Applied Biosystems, Foster City, USA) was employed as recommended by the manufacturer. All cycle sequencing reactions were performed on a Eppendorf Mastercycler gradient thermocycler using an initial denaturation step at 96°C for 5 min, followed by 35 cycles of 96°C for 10 s, 30°C for 20 s, and 60°C for 4 min. Products were purified using the Big Dye Purification Kit (DyeEx 2.0 Spin KIT, Qiagen, Hilden, Germany), dried in a vacuum centrifuge, and resuspended in template suppression reagent (Applied Biosystems, Foster City, USA). Products were then analyzed on a 16*-*capillary 3130xl Genetic Analyzer (Life Technologies GmbH, Darmstadt, Germany). The sequences were formatted and edited using Chromas Lite (Technelysium Pty. Ltd., Australia) and Multalin software (http://multalin.toulouse.inra.fr/multalin/). The appropriate sequences were then compared to sequences listed in the GenBank database using the BLASTn program (http://www.ncbi.nlm.nih.gov/BLAST/). DNA sequences of the native fungal pathogens and the according NCBI hits were then aligned using ClustalW algorithm in MegAlign (DNASTAR Inc., Madison, Wisconsin, USA). Phylogenetic and molecular evolutionary analyses were conducted using MEGA software version 5 using the neighbor-joining (NJ) method (1000 steps) [63]. Species identification was made based on a greater-than-99%-similarity between query and reference sequence according to [28]. The rDNA sequences used for this phylogenetic analysis can be found at the NCBI database under the following GenBank accession numbers: KJ541472, KJ541473, KJ541474, KJ541475, KJ541476, KJ541477, KJ541478, KJ541479, KJ541480, KJ541481, KJ541482, KJ541483, KJ541484, KJ541485, KJ541486, KJ541487, KJ541488, KJ541489, KJ541490, KJ541491, KJ541492, KJ541493 and KJ541494.

### Chemotaxonomical analysis of fungal isolates by UPLC-ToF-MS

Fungal polar metabolite extraction was done using 14 day-old fungi cultures grown on PDA at 25°C in the darkness. The extraction protocol used was adapted from Andersen *et al*. (2005) [64]. Three agar plugs (6 mm diameter) were cut from the central, intermediate and marginal area of the fungal colony. The plugs were extracted with 1.0 mL of ethyl acetate containing 1% (v/v) formic acid by sonication for 60 min. The extract was transferred to a clean 2-mL HPLC vial and evaporated to dryness in a rotary vacuum concentrator (Eppendorf, Wesseling-Berzdorf, Germany). The residue was redissolved ultrasonically in 200 µL of methanol and centrifuged at 16,100 g for 15 min with Eppendorf centrifuge 5415 R (Eppendorf, Wesseling-Berzdorf, Germany). The supernatant was transferred into HPLC vials with insert and used for fungal metabolite analysis with a Ultraperformance LC-ToF-MS system (Agilent Technologies, Böblingen, Germany) using a modified version of a previously described LC-ToF-MS method for *N. attenuata* [65]. Four microliters of the fungal extract were injected onto a C18 column (Acclaim, 2.2 mm particle size, 150 mm 62.1 mm inner diameter (Dionex Corporation, Sunnyvale, USA) and separated using an RSLC system (Dionex). Solvent A was deionized water containing 0.1% (v/v) acetonitrile (Baker, HPLC grade) and 0.05% (v/v) formic acid. Solvent B was acetonitrile and 0.05% (v/v) formic acid. The gradient condition was applied as follows: 0 to 0.5 min 10% B, 0.5 to 6.5 min linear gradient 80% B, 6.5 to 10 min 80% B, and equilibration at 10% B for 3 min. The flow rate was 300 µL min^−1^. Eluted compounds were detected with a MicroToF mass spectrometer (Bruker Daltonic, Bremen, Germany) equipped with an electrospray ionization source in negative ion mode. Instrument settings were as follows: capillary voltage, 4500 V; capillary exit, 130 V; dry gas temperature, 200°C; drying gas flow, 8 liters min^−1^. Mass calibration was performed using sodium formeate clusters (10 mM solution of NaOH in isopropanol/water (50∶50; v/v) containing 0.2% (v/v) formic acid).

The raw data files were converted to netCDF format using the export function of Bruker software (Data Analysis v4.0) and processed using the XCMS package [66] and the R-package CAMERA (http://www.bioconductor.org/biocLite.R) as previously described [65]. Peak detection was performed using the centWave method [66] and the parameter settings ppm = 20, snthresh = 10, peak width = 5 to 20 s. Retention time correction was achieved using the parameter settings minfrac = 1, bw = 60 s, mzwid = 0.1 D, span = 1, and missing  =  extra = 0 [66]. The MetaboAnalyst 2.0 online software (http://www.metaboanalyst.ca/MetaboAnalyst/faces/Home.jsp) was used to perform multivariate analysis (principle component analysis). The data was normalized using Autoscaling. For *Alternaria* sp. Utah 7 metabolic profiling was not possible because of a mistake during extraction. Peak intensities of characteristic ions representing *Alternaria* mycotoxins were determined based on retention times and mass-over-charge values available on a public database [34].

### Morphological characterization of fungal isolates by light microscopy

Mycelium and spore mats of 14 day-old fungal cultures were used for morphological observations under the light microscope (Axio Observer.D1, Carl-Zeiss Jena, Germany). Pictures were taken using AxioVision imaging software at 400-fold magnification. A description of *Fusarium solani* by de Hoog (2000) was used for confirmation of *Fusarium solani* Utah [35], together with reference pictures of its microconidia and macroconidia available online (http://tolweb.org/Sordariomycetes/29050/2008.01.14). *Alternaria* species were characterized using descriptions made by Simmons *et al.* (1999) together with a reference picture of *Alternaria alternata* conidia and conidiophores made by de Hoog (2000) [35], [36].

### Evaluation of fungal isolate pathogenicity using a detached leaf bioassay

For a detached leaf assay plugs of fungal mycelium (3 mm diameter) were cut from the marginal regions of 14 day-old actively growing fungus culture and placed on fully expanded leaves collected from plants at rosette stage (30 day-old plants). The leaves were placed upside down on four layers of moist autoclaved tissue paper in square-shaped petri dishes. Three fungal agar plugs were placed on three distant spots on the abaxial side of a detached *N. attenuata* leaf. All plates were sealed twice with Parafilm to maintain high moisture conditions and kept at 25°C with a 14-hours photoperiod. After incubation for 5 days, the smallest and largest diameter of chlorotic, necrotic and hypersensitive-like lesions was measured using a caliper. Hypersensitive-like lesions as a typical consequence of an incompatible plant interaction with avirulent pathogens leading to disease resistance were distinguished from necrotic lesions caused by other disease-related cell death processes [37]. One-way analysis of variance (ANOVA) and mean value comparison by Fisheŕs LSD post-hoc test was calculated using the SPSS Statistics software version 17.0 (SPSS, Chicago, Illinois, USA). Twelve biological replicates were used for each fungal isolate. Detached leaves incubated with pure PDA were used as control and the agar plug diameter was referred to as “lesion” diameter of controls, although no necrotic and hypersensitive-like lesions occurred, to account for the fact that lesions could only be observed as soon as they exceeded the diameter of the inoculation plug.

### Establishment of pathogen bioassays using the fungal isolates and intact soil-grown *N. attenuata* plants

For the bioassay with entire *N. attenuata* plants, fungal isolates with different virulence (aggressive and moderate isolates) were used with three different spore concentrations (10^5^, 10^6^ and 10^7^ spores mL^−1^). Spores harvested from the surface of 14 day-old fungal cultures were filtered through miracloth (Calibiochem, UK) to remove mycelial fragments and centrifuged at 800 g for 20 min. The pellets were washed twice with 10 mM MgSO_4_ and the spore concentration was adjusted to the desired concentration with 10 mM MgSO_4_ using a Neubauer hemocytometer to count the number of spores under the light microscope. The spore suspensions were used to inoculate *N. attenuata* plants of different age (10 or 20 day-old) using two different infection methods. For the root-dip method, roots were dipped in spore suspension for 30 s and afterwards placed back into the substrate. Spraying with spore suspension was performed homogenously from about 20 cm distance to the plant with approximately 0.3 mL spore suspension per plant. Control plants were dipped into or sprayed with 10 mM MgSO_4_ solution.

## Supporting Information

Figure S1 **Schematic map illustrating changes in the percentage of diseased** ***N. attenuata*** **plants.** The plant number (n) is indicated for each section (labeled I to XIV) of the native *N. attenuata* population. Bluish colors indicate an overall reduction, reddish colors an overall increase in the percentage of diseased plants over time (June 9^th^ 2011 *vs*. May 24^th^ 2011).
(TIF)

Figure S2 **Chemotaxonomy of native fungal pathogen isolates. A**. Dendrogram of native *Fusarium* and *Alternaria* species generated by hierarchical clustering in MetaboAnalyst using Ward’s linkage clustering method based on polar metabolite profiling data (UPLC-ToF-MS in negative ion mode). The numbers on the scale indicate the distance level with relative units. **B**. Two-dimensional distribution of *Alternaria* and *Fusarium* (*F. brachygibbosum* and *F. solani*) isolates according to the two major separating principal components (PCs). **C**. Principal componant analysis of polar metabolite profiles from *Alternaria* isolates. PC1 and PC2 explained together 44.2% of the variance of the samples (the explained variances per component are shown in brackets).
(TIF)

Table S1 **Development of disease symptoms in a native** ***N. attenuata*** **population within a 16 day-time interval.**
(DOC)

Table S2 **Mycotoxins from native** ***Alternaria*** **spp. isolates assigned by mass-over-charge values.**
(DOC)
