# Supplementary figures and images for: Isolating Fungal Pathogens from a Dynamic Disease Outbreak in a Native Plant Population to Establish Plant-Pathogen Bioassays for the Ecological Model Plant Nicotiana attenuata
Source: PLoS One. 2014 Jul 18;9(7):e102915. doi: 10.1371/journal.pone.0102915 (PMC4103856; doi:10.1371/journal.pone.0102915)

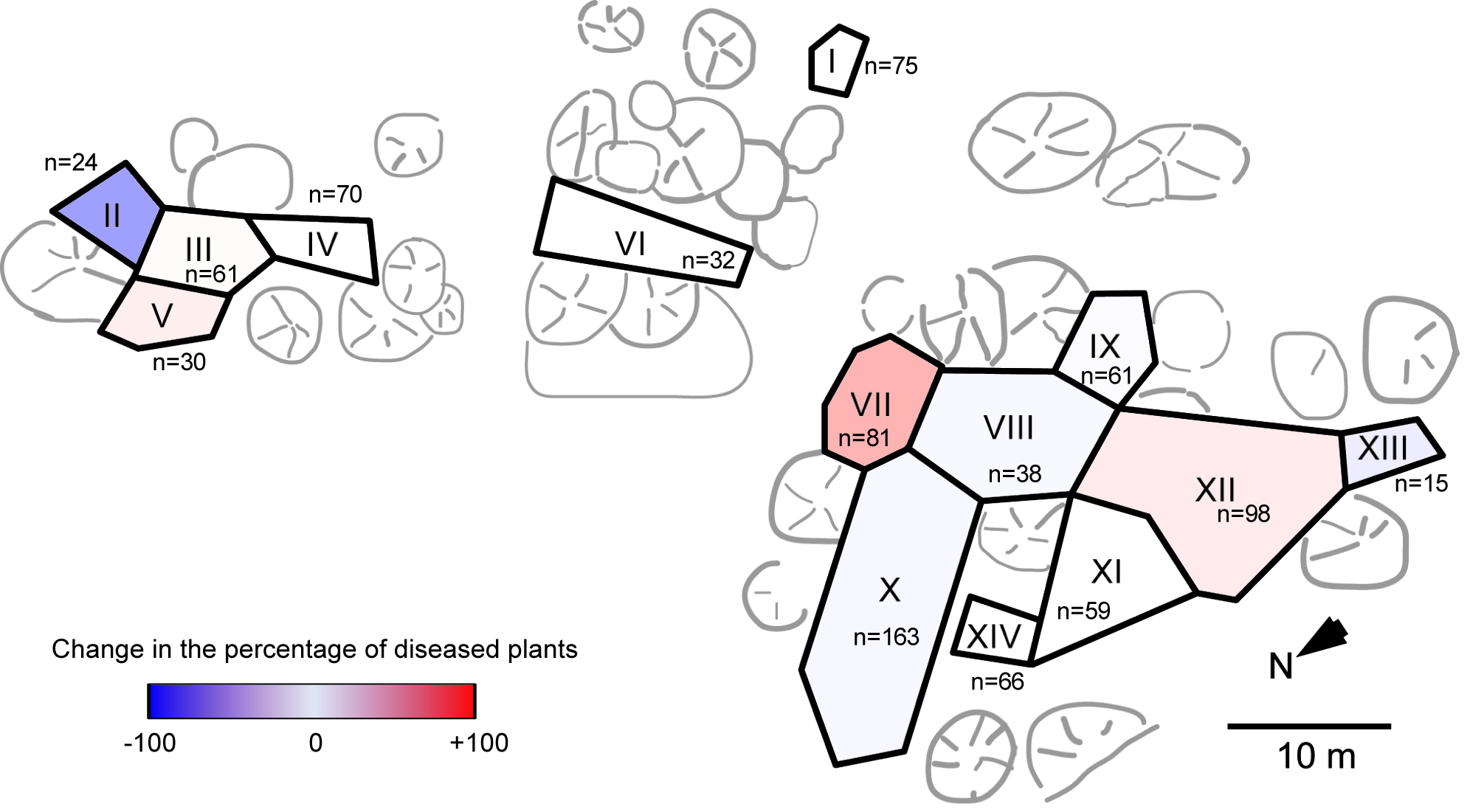

Supplement: Figure S1 — Schematic map illustrating changes in the percentage of diseased N. attenuata plants. The plant number (n) is indicated for each section (labeled I to XIV) of the native N. attenuata population. Bluish colors indicate an overall reduction, reddish colors an overall increase in the percentage of diseased plants over time (June 9th 2011 vs. May 24th 2011). (TIF) [file pone.0102915.s001.tif]

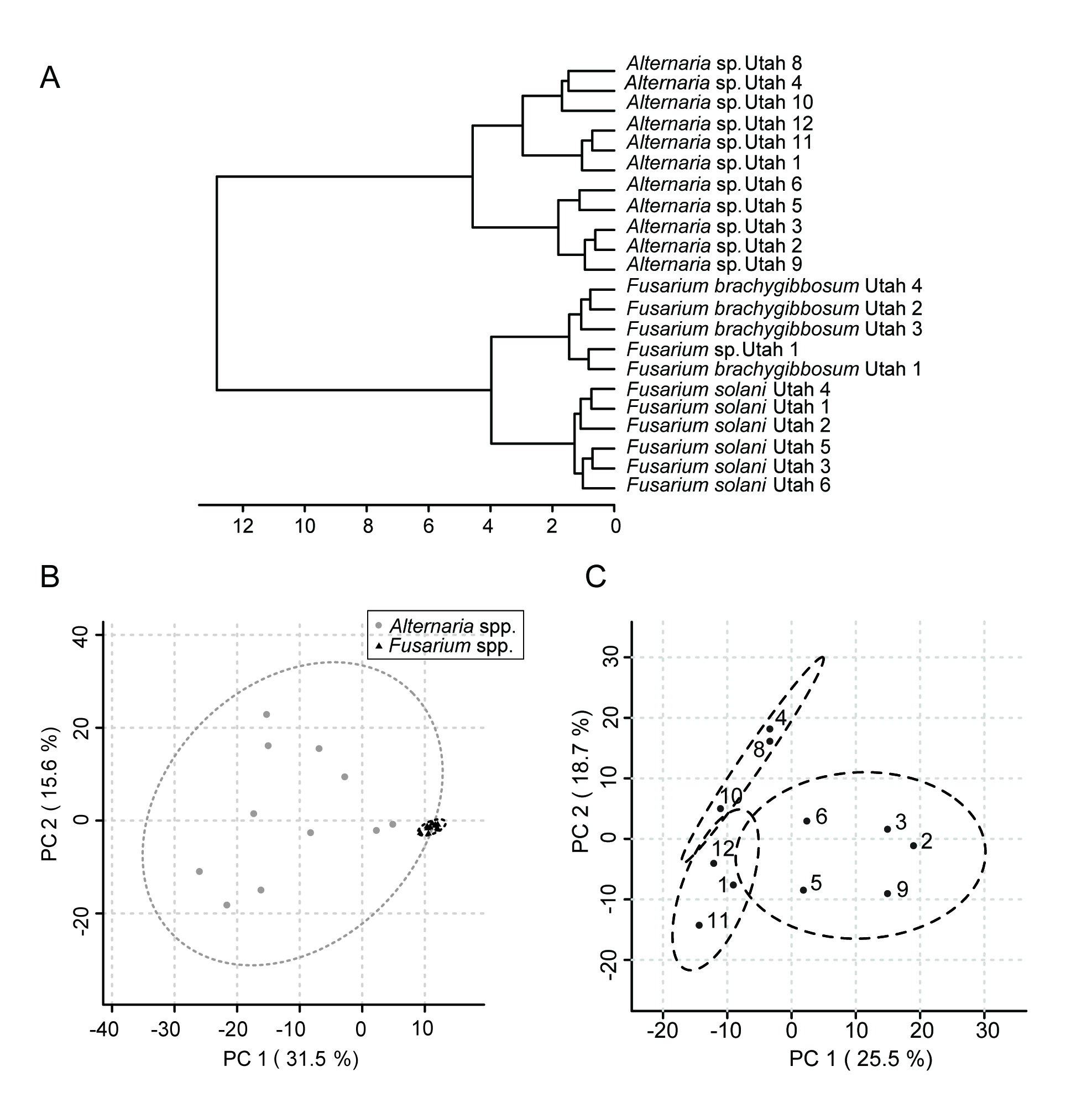

Supplement: Figure S2 — Chemotaxonomy of native fungal pathogen isolates. A. Dendrogram of native Fusarium and Alternaria species generated by hierarchical clustering in MetaboAnalyst using Ward’s linkage clustering method based on polar metabolite profiling data (UPLC-ToF-MS in negative ion mode). The numbers on the scale indicate the distance level with relative units. B. Two-dimensional distribution of Alternaria and Fusarium (F. brachygibbosum and F. solani) isolates according to the two major separating principal components (PCs). C. Principal componant analysis of polar metabolite profiles from Alternaria isolates. PC1 and PC2 explained together 44.2% of the variance of the samples (the explained variances per component are shown in brackets). (TIF) [file pone.0102915.s002.tif]
