# Supplementary material for: Isolating Fungal Pathogens from a Dynamic Disease Outbreak in a Native Plant Population to Establish Plant-Pathogen Bioassays for the Ecological Model Plant Nicotiana attenuata
Source: PLoS One. 2014 Jul 18;9(7):e102915. doi: 10.1371/journal.pone.0102915 (PMC4103856; doi:10.1371/journal.pone.0102915)
Supplement: Table S1 — Development of disease symptoms in a native N. attenuata population within a 16 day-time interval. (DOC) [file pone.0102915.s003.doc]

**Supplemental Table 1. Development of disease symptoms in a native *N. attenuata*** population within a 16 day-time interval.

| **Section** | **I** | **II** | **III** | **IV** | **V** | **VI** | **VII** | **VIII** | **IX** | **X** | **XI** | **XII** | **XIII** | **XIV** | **Sum** |
| --- | --- | --- | --- | --- | --- | --- | --- | --- | --- | --- | --- | --- | --- | --- | --- |
| **Total no. of plants** | 75 | 24 | 61 | 70 | 30 | 32 | 81 | 38 | 61 | 163 | 59 | 98 | 15 | 66 | 873 |
| Diseased at 1^st^ survey | 0 | 10 | 4 | 0 | 20 | 0 | 0 | 1 | 2 | 15 | 4 | 5 | 1 | 0 | 62 |
| Diseased at 2^nd^ survey | 0 | 1 | 5 | 0 | 24 | 0 | 24 | 0 | 1 | 10 | 4 | 13 | 0 | 0 | 82 |
| Fully recovered plants | 0 | 9 | 1 | 0 | 4 | 0 | 0 | 1 | 1 | 13 | 1 | 2 | 1 | 0 | 33 |
| Partly recovered plants | 0 | 0 | 0 | 0 | 2 | 0 | 0 | 0 | 0 | 0 | 0 | 0 | 0 | 0 | 2 |
| Symptom progression | 0 | 1 | 2 | 0 | 11 | 0 | 0 | 0 | 1 | 1 | 3 | 2 | 0 | 0 | 21 |
| Newly diseased | 0 | 0 | 2 | 0 | 8 | 0 | 24 | 0 | 0 | 8 | 1 | 10 | 0 | 0 | 53 |
