# Supplementary material for: Isolating Fungal Pathogens from a Dynamic Disease Outbreak in a Native Plant Population to Establish Plant-Pathogen Bioassays for the Ecological Model Plant Nicotiana attenuata
Source: PLoS One. 2014 Jul 18;9(7):e102915. doi: 10.1371/journal.pone.0102915 (PMC4103856; doi:10.1371/journal.pone.0102915)
Supplement: Table S2 — Mycotoxins from native Alternaria spp. isolates assigned by mass-over-charge values. (DOC) [file pone.0102915.s004.doc]

**Supplemental Table 2. Mycotoxins from native *Alternaria*** spp. isolates assigned by mass-over-charge values.

|  | **Peak intensities of ions representing *Alternaria* mycotoxins [*m/z*]** | | | |
| --- | --- | --- | --- | --- |
|  | **AOH** | **AME** | **Alx I** | **Ten** |
| ***Alternaria* isolate** | **[259.059]** | **[273.075]** | **[351.087]** | **[415.234]** |
| *Alternaria* sp*.* Utah 1 | 275223.8 | 1417.7 | 32567.7 | 143739.7 |
| *Alternaria* sp*.* Utah 2 | 36014.7 | 1213.6 | 401.4 | 41718.4 |
| *Alternaria* sp*.* Utah 3 | 685327.6 | 1446.7 | 10401.8 | 188794.7 |
| *Alternaria* sp*.* Utah 4 | 416540.2 | 2017.9 | 3851.7 | 3979.8 |
| *Alternaria* sp*.* Utah 5 | 32121.9 | 1227.9 | 404395.4 | 16496.7 |
| *Alternaria* sp. Utah 6 | 37545.0 | 1756.5 | 342584.3 | 12643.5 |
| *Alternaria* sp. Utah 8 | 1057.3 | 2808.8 | 13295.6 | 0.889 |
| *Alternaria* sp. Utah 9 | 102667.5 | 2035.5 | 48820.2 | 2483.3 |
| *Alternaria* sp. Utah 10 | 125329.2 | 1854.1 | 64100.2 | 22212.1 |
| *Alternaria* sp. Utah 11 | 782.4 | 0.889 | 197.6 | 202.7 |
| *Alternaria* sp*.* Utah 12 | 89926.6 | 434.0 | 194645.8 | 13992.6 |

**AOH**: Alternariol; **AME**: Alternariol monomethyl ether; **Alx I**: Altertoxin I; **Ten**: Tentoxin
